# Identifying Prognostic Factors Implicated in Chronic Functional Abdominal Pain in Children: A Systematic Review

**DOI:** 10.14740/gr2141

**Published:** 2026-08-26

**Authors:** Hannah Richardson, Ruth Bland, Kirsty Gray

**Affiliations:** a Department of Paediatrics, Queen Elizabeth University Hospital, Glasgow, UK; b Centre for Virus Research, University of Glasgow, Glasgow, UK

**Keywords:** Pediatric functional abdominal pain, Functional gastrointestinal disorder, Prognostic indicators, Psychosocial factors

## Abstract

Pediatric functional abdominal pain can place a significant burden on children and their families. Recent guidance has identified the benefits of hypnotherapy and cognitive behavioral therapy as management options; however, pharmacological approaches have only low certainty evidence. The aim of this study was to identify prognostic factors of chronic pain and risk of developing additional functional gastrointestinal disorders, among children with functional abdominal pain. Potential therapies could be targeted towards a population of children identified at higher risk of developing chronic abdominal pain. Relevant studies were identified through a search undertaken on four databases: Ovid MEDLINE, Embase, APA PsychInfo, and the Cochrane library, using a combination of MeSH terms and free text. Two independent reviewers screened results of this search utilizing set eligibility criteria, summarizing the characteristics of each included study according to a standardized table and assessing for risk of bias. Eight prospective cohort studies were included in the final review, with a range of duration of follow-up between 6 months to 15 years. Later age of onset of pain appeared to predict chronicity of pain, however not the development of an additional functional gastrointestinal disorder. The use of pain profiles, combining gastrointestinal and non-gastrointestinal symptoms with intensity of pain, appeared to be particularly useful for predicting later development of a functional gastrointestinal disorder. Comparison between the studies was limited due to the heterogeneity of the patient profiles, prognostic factors and outcomes that were assessed. Future research should be directed to larger, standardized studies to assess the wide range of prognostic factors which may play a significant role in chronic functional abdominal pain.

## Introduction

Functional abdominal pain (FAP) is a common condition in the pediatric population and can be a significant cause of anxiety among patients and families, as well as a major reason for school absence [1]. The estimated worldwide prevalence of FAP disorders is 11.7%, with no statistically significant difference demonstrated between the continents. There has consistently been an underrepresentation of studies conducted in Africa, with only two studies out of a total of 66 included in a recent meta-analysis [2].

The incidence is much higher amongst girls, and there is an association with psychosocial factors including depression, anxiety and traumatic life events [3, 4]. Most children experience resolution of their abdominal pain; however, a recent review demonstrated that 29.1% of children can develop chronic abdominal pain, persisting on average 5 years after initial diagnosis [5].

The pathogenesis of FAP remains unclear, and the diagnosis largely relies on the exclusion of other organic causes for the pain. This may be through history and examination alone, with a recent review demonstrating no improvement in prognosis when additional medical investigations were undertaken [5]. Since 1999, the ROME criteria were introduced to aid the diagnosis of functional gastrointestinal disorders (FGID) in children [6, 7]. Updated in 2016, the ROME IV defines functional abdominal pain–not otherwise specified (FAP-NOS) as chronic or episodic abdominal pain, not fully explained by an organic cause or meeting the criteria for another FGID. The pain must be present at least four times per month for at least 2 months prior to a diagnosis. Worldwide data regarding epidemiology of FAP disorders has utilized different ROME criteria for definition with resulting differences in prevalence (13.2% for ROME III compared to 9% for ROME IV) [2].

A recent review by the European and North American Societies for Pediatric Gastroenterology, Hepatology, and Nutrition has provided recommendations on pharmacological and non-pharmacological approaches to FAP and irritable bowel syndrome (IBS) based on the level of evidence available. Based on available evidence, the strongest recommendations were provided for hypnotherapy and cognitive behavioral therapy (CBT). Percutaneous electrical nerve field stimulation has also been recommended as a new emerging therapy. With regard to hypnotherapy, eight studies were included in the review, either comparing hypnotherapy with a control intervention or assessing the outcomes between the different forms of provision. Randomized controlled trials have shown a significant improvement in outcomes with hypnotherapy in pediatric patients compared to standard care, but that no specific treatment module is preferential (gut-directed, in-person, self-directed) and that any could be considered. Within the adult population, gut-directed hypnotherapy has been shown to have a notable effect on sensorimotor function and modulating brain-gut pain pathways and has been recommended for use in severe refractory FGID, while group CBT has been demonstrated to have significant benefit within patients with refractory IBS [8, 9].

As seen in prior reviews, there was limited evidence for pharmacological treatments; however, some can be considered on an individual patient basis, for example: multi-strain probiotics, enteric-coated peppermint capsules, amitriptyline, domperidone and cyproheptadine. The guidance highlights the importance of providing the patients with education regarding their diagnosis and advise caution with the use of dietary modifications or over-the-counter analgesia for chronic pain [10, 11].

The aim of this review is to identify factors which may predispose children with FAP to a higher risk of chronic abdominal pain, FGID or pain-related disability. In this way, treatments could be targeted to this cohort of children identified at risk, or to addressing the prognostic factors themselves.

## Methods

### Search strategy

A literature search was undertaken on April 19, 2024, using Ovid MEDLINE, Embase, APA PsychInfo, and the Cochrane library (Supplementary Material 1, gr.elmerpub.com). Searches were limited to the past 15 years and those published in the English language.

Following removal of duplicates, two independent authors utilized set eligibility criteria to select the studies for review and undertake data extraction. This review adhered to the Preferred Reporting Items for Systematic Reviews and Meta-Analyses (PRISMA) 2020 guidance.

### Eligibility criteria

#### Inclusion criteria

The selected population group for this review were children up to the age of 19, with FAP of non-organic etiology. Studies were included if their outcomes included either persistence of the abdominal pain, pain-related disability or development of another FGID beyond 6 months.

#### Exclusion criteria

Specific exclusion criteria for this review included studies not published in English or those not involving human participants. Studies were excluded if they assessed response to any intervention out with standard symptom management, or if they assessed children with an organic disease. Unpublished studies, conference abstracts, case series and reports were excluded. Due to the likelihood of a difference between etiologies or prognostic factors for different FGIDs, studies focusing solely on functional dyspepsia, IBS or functional constipation were excluded.

### Data extraction and assessment of bias

Two authors independently undertook data extraction and assessment of bias. Data were extracted using a standardized form, outlining the target population, number of participants, outcomes measured, authors’ conclusions and any limitations identified.

Risk of bias was assessed using the Newcastle-Ottawa Scale (NOS) for non-randomized cohort studies. Stars are awarded following set guidance with respect to three domains: selection, comparability, and outcome [12]. These are then converted to a previously agreed rating of poor, fair or good quality as per Agency for Healthcare Research and Quality (AHRQ). These were defined as follows: (1) good quality: three or four stars in selection domain, and one or two stars in comparability domain, and two or three stars in outcome/exposure domain; (2) fair quality: two stars in selection domain, and one or two stars in comparability domain, and two or three stars in outcome/exposure domain; (3) poor quality: zero or one star in selection domain, or zero stars in comparability domain, or zero or one star in outcome/exposure domain [13].

Where there was disagreement between results, particularly through use of the NOS for assessment of selection bias, opinion was sought from a third independent reviewer.

## Results

**Figure 1:**
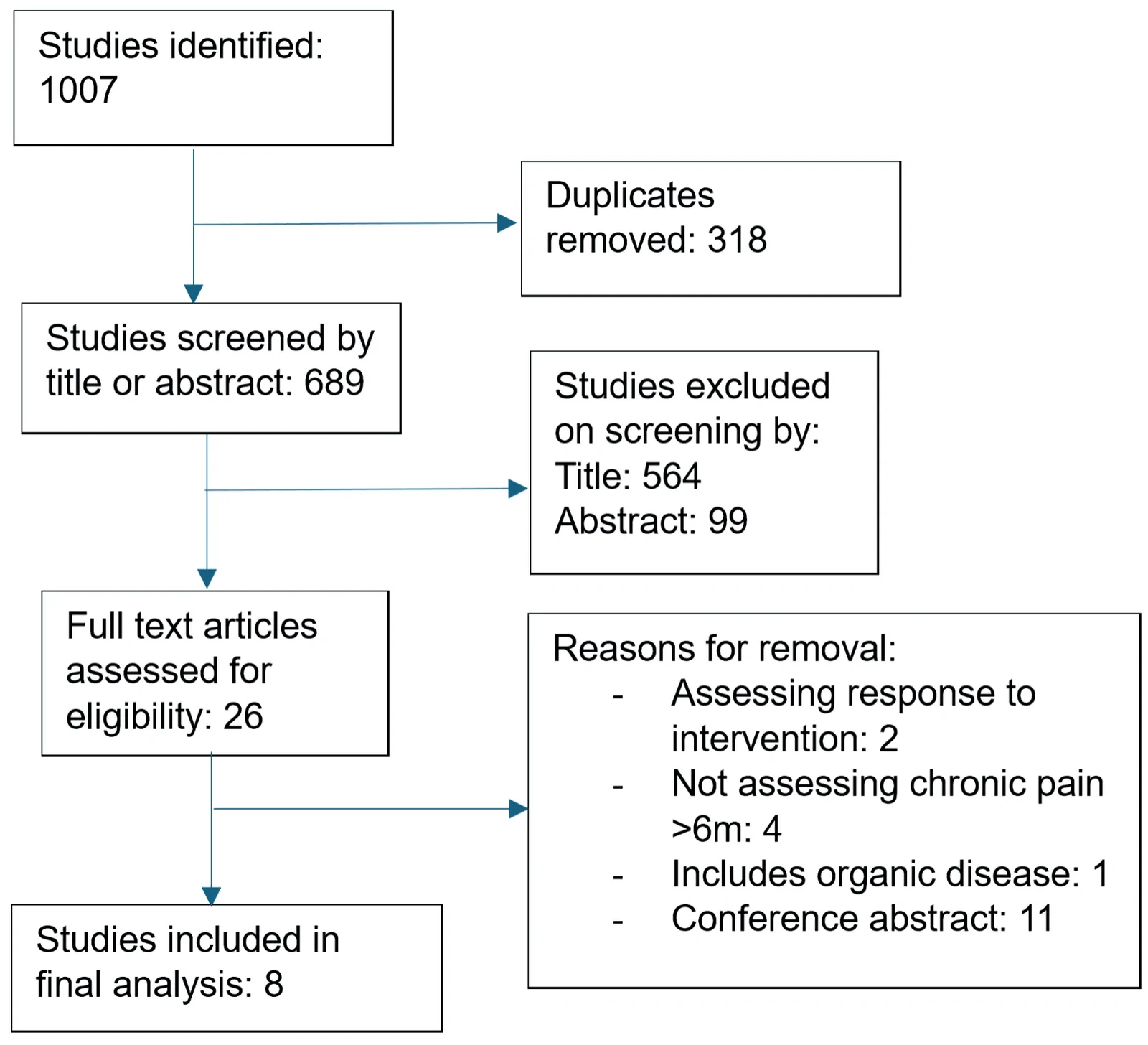
PRISMA flowchart. PRISMA: Preferred Reporting Items for Systematic Reviews and Meta-Analyses.

The initial search identified 689 studies once duplicates were removed. The majority of these were removed by screening the title or abstract using eligibility criteria. Twenty-six articles were assessed in full and screened as follows: 11 conference abstracts were removed; one study was excluded as it was a follow-up on the comparison between the use of drotaverine versus placebo [14], and another was excluded because it compared the use of two different forms of behavioral therapy [15]. Four studies did not assess chronic pain or FGID beyond 6 months [16–19], and one study did not exclude children with an organic cause for their abdominal pain [20]. Eight studies were included in the final review (Fig. 1).

### Characteristics of included studies

The characteristics of the included studies are summarized in Table 1 [21–28]. All the studies were prospective cohort in nature.

**Table 1:** Characteristics of Included Studies

Authors, year	Aim of study	Type of study	Number of patients	Target population	Outcome measure	Summary of results	Limitations
Sjolund et al, 2021 [21]	To assess the natural history of recurrent childhood abdominal pain from early childhood through adolescence	Prospective population-based birth cohort study	2,455, from original cohort of 4,089	Children born 1994–1996 in Stockholm, Sweden, followed from infancy to 16 years	RAP, AP-FGID and IBS at 16 years by parental/child questionnaires scored against Rome III criteria	Recurrent abdominal pain age 12 years is predictive of RAP/FGID at age 16 years	Recurrent abdominal pain at age 1–2 years defined as parent-reported colic. Outcome largely based on questionnaire/self-report; around 60% cohort retention with potential selection bias.
Cunningham et al, 2017 [23]	To identify link between pain intensity, pain-related disability and anxiety with pain-related disability at 6 months	Prospective cohort study	99 at baseline, 64 at follow-up	Pediatric FGID patients (ages 8–18, Rome III, gastroenterologist-diagnosed) at a large Midwestern children’s hospital, USA	Functional Disability Inventory-Child version (FDI-C, self-report) at 6 months	Patients categorized by number of risk factors (0–3). The presence of 2+ risk factors predicted pain-related disability at 6 months	Little differentiation between FAP/IBS/FD. Short duration follow-up. Small number of participants with 35% lost to follow-up
Russell et al, 2017 [22]	To identify the link between nausea and long-term morbidity in pediatric patients with FAP	Prospective cohort study	871 at baseline, 392 at followed-up	Pediatric FAP patients (8–17 years) seen in a pediatric GI clinic, USA	GI, non-GI symptoms, depression, functional disability > 4 years later	Nausea is associated with higher risk of depression, anxiety, GI and somatic symptoms 4 years later	No assessment of outcomes at start of assessment (e.g., correlation of nausea with current anxiety/non-somatic symptoms). Approx 52% follow-up; age difference between groups wer not adjusted for.
Czyzewski et al, 2016 [28]	To assess whether anxiety or pain–stooling relations predict long-term maintenance of abdominal pain	Prospective cohort study	76	Children aged 7–10 years with FAP recruited from primary and tertiary care, USA	Parent-reported abdominal pain frequency 18–24 months later	Anxiety not predictive of persistence of pain. IBS symptoms are predictive	Overlap between IBS and FAP in intervention group. Small sample. No adjustment for confounders.
Horst et al, 2014 [25]	Relation between GI symptoms, extra-intestinal somatic symptoms and depressive symptoms on likelihood of FGID at follow-up	Prospective cohort study	760 at baseline, 392 at followed-up	8–16Y with recurrent chronic abdominal pain (Apley criteria), at a single tertiary pediatric GI clinic, USA	ROME III FGID (IBS, FD, FAPS, abdominal migraine) > 3 years later	41% met ROME III criteria at follow-up, mostly IBS. Extraintestinal somatic and depressive symptoms were predictive. Severity of abdominal pain, age and gender were not.	ROME III used for follow-up but not for initial diagnosis. Some of GI symptoms at exposure may indicate a diagnosis of IBS/FD. Tertiary care sample may limit generalizability, interim treatment was not captured.
Walker et al, 2012 [26]	Identify childhood FAP profiles and test whether they predict FGID, psychiatric disorder and central sensitization in later life	Prospective cohort study	Baseline 843, 760 eligible for follow-up. 379 followed-up, 211 for lab subset	Consecutive new pediatric FAP patients at tertiary pediatric clinic, USA, average age 12 years	ROME III FGID and chronic pain (PPQ), DSM IV anxiety/depressive disorder	Three profiles identified. Association between high pain dysfunctional type and FGID at follow-up	Approx 50% attrition; no baseline psychiatric/chronic-pain assessment; tertiary-care sample may limit generalizability.
Bonilla et al, 2011 [24]	Investigate whether obesity predicts persistence of pain and disability at long-term follow-up of FGIDs	Prospective cohort study	Identified 301, 188 enrolled and followed-up	Children, mean age 13.3 years, with ROME II FAP/IBS/FD, single pediatric GI division, USA	Persistence of pain, pain intensity/frequency and school absenteeism at follow-up 12–15 months later	61.7% had persistent pain at follow-up. Obesity is associated with poor long-term outcome and disability	Included IBS/FAP/FD (not much differentiation). Diet was not controlled. Unadjusted for age, gender, baseline severity. No range for age given; however, all participants were recruited from pediatric clinic.
Helgeland et al, 2011 [27]	Whether pediatric FAP outpatients have more somatic/mental-health symptoms than a population sample, and whether child and maternal symptoms predict persistent abdominal pain and disability at follow-up	Prospective cohort study	Baseline 94, follow-up 88	8–15 years old, with FAP; four general pediatric outpatient clinics, Norway	Self-reported abdominal pain index and functional disability inventory at 6–9 months	Patients had far more somatic and emotional symptoms than school children. Older age at baseline and peer problems were associated with persistent abdominal pain. Older age, emotional symptoms, prosocial behavior and maternal somatic symptoms predicted disability.	Short follow-up, self-reported outcomes. Child emotional symptoms only by mother/teacher.

RAP: recurrent abdominal pain; FAP: functional abdominal pain; IBS: irritable bowel syndrome; FGID: functional gastrointestinal disorder; FD: functional dyspepsia; GI: gastrointestinal.

#### Population

The studies varied greatly in terms of size of population, with the smallest study consisting of 64 participants and the largest population-based study assessing a total of 2,455 [21]. In all the studies, there was a higher proportion of females, in general between 60–70% of the cohort. Most of the studies recruited participants from tertiary gastroenterology services [22–26], with one study recruiting solely from primary care [27] and one study from a mixture of primary and tertiary services [9]. The majority of the studies recruited participants age 8 years and older to allow reliable self-report of symptoms. One study did not provide the exact age range; however, all the patients had been recruited from a pediatric gastroenterology department. This same study recruited their cohort based on ROME II criteria [24]. None of the other studies utilized ROME criteria to define FAP in their initial population selection, instead recurrent abdominal pain (RAP) was clearly defined by set criteria, ensuring to exclude children and adolescents with an organic cause for their symptoms.

#### Exposure

There was great heterogeneity between definitions of FAP utilized by the individual studies and the prognostic factors assessed. This made generalizability between results impossible. Only one study recruited their cohort based on ROME criteria, while the remainder defined RAP using set criteria. The range of prognostic factors included psychosocial and gastrointestinal features associated with the pain.

#### Outcome

The outcome for most of the studies was either chronic RAP, a diagnosis of an FGID, or pain-related disability. The shortest length of follow-up for outcomes was 6 months [23, 27] and the longest 15 years [21].

### Risk of bias summary

Utilizing the NOS scale and appropriate AHRQ standards, most studies met the criteria for fair quality (Table 2) [21–28]. Three of the studies met the criteria for a poor standard and high risk of bias. There was a greater potential for attrition bias, with a large loss of cohort to follow-up with no clear explanation or description of demographics in this group [23]. For Czyzewski et al, there was a recognition that children with lower pain frequency were more likely to be lost to follow-up, impacting the generalizability of results [28]. The only study to meet a good quality standard was that by Sjolund et al, which differed from the other studies as it was a birth cohort study, therefore the outcome of interest was not present at the start [21]. Unlike the other studies, Bonilla et al [24] used ROME criteria to define their initial cohort, while a few of the studies used these criteria to identify their outcomes. There is a reasonable risk of reporting bias within the current systematic review: both from the 11 studies which had not been published in full text, as well as others that were potentially not found due to lack of publication. A request was made to the authors of the conference abstracts for access to the full paper; however, these did not receive a response.

**Table 2:** Risk of Bias Assessment

Study	Selection^a^	Comparability	Outcome	Quality assessment^b^
Sjolund et al, 2021 [21]	✰✰✰	✰✰	✰✰	Good
Cunningham et al, 2017 [23]	✰✰✰	✰	✰	Poor
Russell et al, 2017 [22]	✰✰	✰	✰✰	Fair
Czyzewski et al, 2016 [28]	✰✰		✰	Poor
Horst et al, 2014 [25]	✰✰	✰✰	✰✰✰	Fair
Walker et al, 2012 [26]	✰✰	✰✰	✰✰✰	Fair
Bonilla et al, 2011 [24]	✰✰✰		✰✰	Poor
Helgeland et al, 2011 [27]	✰✰	✰✰	✰✰	Fair

^a^Assessment using Newcastle-Ottawa Quality assessment scale. ^b^Conversion to AHRQ standards (good quality: three or four stars in selection domain, and one or two stars in comparability domain, and two or three stars in outcome/exposure domain; fair quality: two stars in selection domain, and one or two stars in comparability domain, and two or three stars in outcome/exposure domain; poor quality: zero or one star in selection domain, or zero stars in comparability domain, or zero or one star in outcome/exposure domain)

### Results of individual studies

Several studies controlled for age and gender due to their potential effect as confounding factors. Two studies assessed the effect of age at initial development of abdominal pain on possible persistence of the pain. Sjolund et al [21] found that there was a significant association between RAP at 12 years and ongoing RAP at 16 years, whereas there was no association with early childhood colic age 1–2. There were no other points of follow-up assessed to clarify the relationship with age [21]. Helgeland et al found a significant association between older age at baseline of abdominal pain and higher level of pain and pain-related disability at follow-up 6 to 9 months later. They also found an association with female gender and higher levels of pain at follow-up; however, this was not statistically significant [27]. Horst et al did not find a significant association between age or gender as predictors of FGID later in life [25].

Cunningham et al found that there was a combined predictive effect between anxiety, pain intensity and functional disability at baseline with functional disability 6 months later; however, they found no significant association with one of these prognostic factors alone [23]. Another study did not find a significant association between anxiety and pain frequency at follow-up [28]. Horst et al identified depressive symptoms as a predictor of development of an FGID, in particular IBS and functional dyspepsia. Other independent prognostic factors identified were the severity of gastrointestinal symptoms and also the number of extra-intestinal somatic symptoms at baseline with presence of an FGID at follow-up 5 to 15 years later [25]. One study defined those with RAP as one of three profiles [26]. They found an association between those regarded as high pain dysfunctional (higher pain intensity, more gastrointestinal and non-gastrointestinal symptoms, increased levels of pain catastrophizing) with later development of an FGID.

Several studies identified gastrointestinal symptoms as predictors of later pain severity and development of an FGID, both as a measure of cumulative symptoms and the presence of individual symptoms such as nausea or pain–stooling relations alone [22, 25, 28].

Only one study assessed body mass index (BMI) as a potential factor in RAP. Bonilla et al found a significant association between obesity and frequency and intensity of pain at follow-up 12–15 months later [24].

## Discussion

### What this study adds

This review adds to the limited knowledge of prognostic factors associated with chronic FAP in childhood. Several potential factors have been identified: in particular older age at onset, psychosocial symptoms (e.g., anxiety or pain catastrophizing), as well as a range of gastrointestinal symptoms. A few studies highlighted the combined effect of several prognostic factors, when one factor alone is not significantly associated with outcome. One study attributed patient profiles to combine multiple gastrointestinal, non-gastrointestinal and psychological prognostic factors and demonstrated differing outcomes on FAP [26].

### Areas of bias and potential limitations

All the studies in this review were prospective in nature, and generally there was a low loss to follow-up. This minimizes the potential for recall or attrition bias. They also cover a range in length of follow-up from 6 months to 15 years, therefore giving a broad spectrum for the outcome of RAP. There was a range in outcomes assessed between studies, including chronic abdominal pain, development of an FGID or pain-related disability. Some studies did use ROME criteria to define outcome. There were no identified competing interests in the process of this review.

As previously discussed in the literature, the conclusions drawn by this review are limited by the relatively low number of studies which met inclusion and exclusion criteria, the small size of the studies and their heterogenous nature in terms of exposure and outcomes measured [29]. Despite the availability of set ROME criteria for the definition of FAP, only one study utilized these criteria in their definition at recruitment, and they failed to use the most recent version [24]. Generally, the authors adopted pre-defined criteria for RAP and clearly stated these in their papers. Sjolund et al [21] encompassed parent-reported “colic” within the RAP category at age 1–2 years and did not specify what features met the criteria for the term colic, aside from parent-report. This was then compared to reported RAP at 12 years. These two measures are very subjective and not necessarily comparable.

Pain is a subjective measure for both exposure and outcome, and all the studies relied on self-report. This impacted the bias assessment in “outcome” for all but one of the studies, due to the nature of the pain measurement. Another aspect of the bias assessment affected by this was within the “selection” criteria, since the outcome of interest (abdominal pain) was present at the start of the study. This shifted most of the studies into the “fair” category and potentially highlights that an adapted form of the bias assessment could be required. With respect to this review, the exposure and outcomes identified did not require a scale of pain, rather its presence or absence, and therefore self-reporting was felt likely to be less significantly biased. Previous studies have demonstrated that patients do not tend to over-report their pain and that their reports of chronic pain remain relatively stable with little evidence of recall bias [30, 31].

Several of the studies recruited patients from tertiary gastroenterology clinics and this has the potential to influence the generalizability of the cohort to the general population of pediatric FAP. It is proposed that children requiring management under tertiary services may have more significant abdominal pain or health-related anxieties. A 2008 review, however, has previously demonstrated no significant difference in prognosis for RAP recruited from secondary or tertiary services [5].

Although all the studies considered confounding factors to some degree, very few of them assessed potential therapies the patients had received prior to follow-up, and this could have potentially significant effects on the outcome. One study found a significant association between BMI and prognosis of RAP [24]. None of the other studies considered this as a potential confounding factor, and a recent analysis has demonstrated no such association [2].

A particular limitation identified in this review was that three out of the eight studies assessed children from overlapping cohorts originally recruited in previous studies during the 1990s and 2000s [22, 25, 26]. There was also no discussion of the proportion of the original databases who were then recruited for these subsequent studies, nor the demographics of those not included.

### Areas for future research

It has been highlighted that larger, more rigorous studies are required into this topic. In particular, the adoption of ROME IV criteria for recruitment and measurement of outcome would make comparison between studies much more reliable. Soon-to-be introduced, the ROME V criteria, aims to differentiate between research and clinical diagnostic criteria, in particular specifying thresholds and endpoints. Focus should be placed on the severity of pain at onset and follow-up through the use of comparable scales (e.g., the visual analogue scale). The use of a reliable change index helps identify a statistically significant difference [6, 32]. This review has implications in the management of childhood FAP, allowing the identification of a cohort of children at higher risk of chronic abdominal pain. Services and potential therapies can therefore be targeted at this population to modify psychosocial symptoms, including anxiety and pain catastrophizing.

As previously discussed, the evidence surrounding treatment of FAP is limited; however, non-pharmacological treatment approaches, particularly CBT and hypnotherapy, should be considered. Some pharmacological options may be considered on an individual basis. Popular strategies utilized by parents include dietary changes, particularly high-fiber diets or gluten and dairy exclusion. These have shown no statistically significant evidence of benefit and may cause harm through nutrient deficiencies. A diet low in fermentable oligosaccharides, disaccharides, monosaccharides and polyols has been shown to improve symptoms in IBS; however, there is a lack of evidence for this treatment in FAP [33]. Larger, more robust randomized controlled trials are required to quantify the benefit that these psychological and pharmacological approaches can provide.

## Conclusions

This review demonstrates a link between gastrointestinal symptoms, psychosocial factors and later age of onset of pediatric FAP with persistence of symptoms. This may be through an interplay of a variety of different prognostic factors, rather than solely relying on one factor alone. There may be a greater number of prognostic factors yet to be identified, out with those assessed in the small selection of studies available. This review suggests that larger studies, utilizing ROME criteria, are required in this field in order to highlight a cohort of children and adolescents at highest risk of chronic pain and disability. Through identification of a specific high-risk profile in pediatric patients with FAP, thorough assessment and early non-pharmacological interventions may by directed appropriately to improve the poor outcomes in this group.

## Supplementary Material

Suppl 1 Search strategy using Ovid MEDLINE, Embase, APA PsychInfo, and the Cochrane library.

## Data Availability

The authors declare that data supporting the findings of this study are available within the article.
