# Supplementary material for: Identifying Prognostic Factors Implicated in Chronic Functional Abdominal Pain in Children: A Systematic Review
Source: Gastroenterology Res. 2026 Aug 26;19(4):175–83. doi: 10.14740/gr2141 (PMC13568790; doi:10.14740/gr2141)
Supplement: Supplementary file 1 [file gr-19-04-175-s001.docx]

**Suppl 1.** Search strategy using Ovid MEDLINE, Embase, APA PsychInfo, and the Cochrane library.

**MEDLINE search strategy** (1946 to 2024 April 19)

| # | Search | Results |
| --- | --- | --- |
| 1 | exp Infant/ or exp Child/ or Adolescent/ | 4038852 |
| 2 | exp Pediatrics/ | 63280 |
| 3 | (child* or infant* or adolesc* or teen* or paediatr* or pediatr*).ti,ab,kf. | 2479505 |
| 4 | 1 or 2 or 3 | 4676225 |
| 5 | exp Prognosis/ | 1967374 |
| 6 | (predict* or prognos* or outcome*).ti,ab,kf. | 4725633 |
| 7 | 5 or 6 | 5671656 |
| 8 | (functional abdominal pain or FAP or FAPS or FAPD).ti,ab,kf. | 7652 |
| 9 | 4 and 7 and 8 | 537 |
| 10 | limit 9 to (english language and yr="2009 -Current") | 370 |

**Embase search strategy** (1974 to 2024 April 19)

| # | Search | Results |
| --- | --- | --- |
| 1 | exp Infant/ or exp Child/ or Adolescent/ | 4071750 |
| 2 | exp Pediatrics/ | 130065 |
| 3 | (child* or infant* or adolesc* or teen* or paediatr* or pediatr*).ti,ab,kf. | 3090578 |
| 4 | 1 or 2 or 3 | 4891206 |
| 5 | exp Prognosis/ | 948503 |
| 6 | (predict* or prognos* or outcome*).ti,ab,kf. | 6629713 |
| 7 | 5 or 6 | 6802596 |
| 8 | (functional abdominal pain or FAP or FAPS or FAPD).ti,ab,kf. | 12513 |
| 9 | 4 and 7 and 8 | 674 |
| 10 | limit 9 to (english language and yr="2009 -Current") | 544 |

**APA PsychInfo search strategy (**1806 to April Week 3 2024)

| # | Search | Results |
| --- | --- | --- |
| 1 | (child* or infant* or adolesc* or teen* or paediatr* or pediatr*).ab,ag,id,ti. | 1321100 |
| 2 | exp Pediatrics/ | 38390 |
| 3 | exp Prognosis/ | 11007 |
| 4 | (predict* or prognos* or outcome*).ab,id,ti. | 986862 |
| 5 | (functional abdominal pain or FAP or FAPS or FAPD).ab,id,ti. | 551 |
| 6 | 1 or 2 | 1325029 |
| 7 | 3 or 4 | 987245 |
| 8 | 5 and 6 and 7 | 70 |
| 9 | limit 8 to (english language and yr="2009 -Current") | 61 |

**Cochrane Library search strategy** (Jan 2009 to Apr 2024)

| Search fields | | Search terms |
| --- | --- | --- |
|  | Title Abstract Keyword | child* or infant or adolesc* or teen* or paediatric* or pediatr* or pediatrics |
| AND | Title Abstract Keyword | predict* or prognos* or outcome* or prognosis |
| AND | Title Abstract Keyword | functional abdominal pain or FAP or FAPS or FAPD |
| Total studies = 32, all in English | | |
